# Evaluating the Effectiveness of a Comprehensive Interventional Package on the Quality of Life of Children With Asthma

**DOI:** 10.7759/cureus.76135

**Published:** 2024-12-21

**Authors:** Deepthi Chakravarthy, Shuba Sankaranarayanan, Anita David, Nirmala V

**Affiliations:** 1 Child Health Nursing, College of Nursing, Muslim Educational Society (MES) Medical College Hospital, Perinthalmanna, IND; 2 Pediatric Medicine, Sri Ramachandra Institute of Higher Education and Research, Chennai, IND; 3 Pediatric Nursing, Sri Ramachandra Institute of Higher Education and Research, Chennai, IND; 4 College of Nursing, Muslim Educational Society (MES) Medical College Hospital, Perinthalmanna, IND

**Keywords:** asthma symptom control, asthmatic children, breathing exercise, bronchial asthma, health-related quality of life

## Abstract

Introduction: Asthma is a chronic lung disease that negatively affects children's quality of life (QoL). Improving QoL is a key outcome in managing children with asthma.

Objectives: The objective of the study was to evaluate the effectiveness of a comprehensive interventional package on QoL among asthmatic children attending an asthma clinic at a selected hospital.

Hypothesis: The study hypothesizes that the experimental group will show significant improvement in QoL compared with the control group.

Method: This quantitative experimental research study was conducted among 94 asthmatic children aged 10-17 years presenting at a pediatric asthma clinic in a selected hospital in Malappuram, Kerala. Participants who met the inclusion criteria were randomized in a single-blind study and allocated to either a control group (N = 47) or an experimental group (N = 47) using allocation concealment methods. Data were collected using three tools: the Asthma Knowledge Questionnaire, the Mini Pediatric Asthma QoL Questionnaire, and the Asthma Control Questionnaire. The control group received no comprehensive interventional package and continued with their medical treatment, while the experimental group received a comprehensive interventional package that included asthma education and Buteyko breathing exercises along with routine medical treatment. Both groups were assessed four times at four-week intervals using the same tools.

Results: Effectiveness was assessed by using the statistical analysis of repeated measures of analysis of variance. The comprehensive interventional package group showed a significant improvement (p < 0.001) in QoL compared with the control group.

Conclusion: The comprehensive interventional package, which includes both asthma education and breathing exercises, can significantly improve asthma knowledge, symptom control, and QoL in children with asthma. This study revealed that improving the understanding regarding the asthma and practicing a breathing exercise regularly along with routine medical treatment would help the children to reduce asthma burden.

## Introduction

An estimated 1%-18% of people worldwide suffer from asthma, a persistent respiratory condition. It is characterized by recurrent episodes of coughing, chest tightness, wheezing, and shortness of breath. The prevalence and severity of asthma vary greatly [1].

Children are disproportionately affected by respiratory disorders, which can lead to airway obstruction or respiratory failure. Bronchial asthma is a long-term illness. Characterized by rapid breathing, the term "asthma" comes from the Greek word "Az-ma," meaning "to breathe with open mouth" or "rapid breathing [2].

The World Health Organization estimates that 235 million people worldwide are affected by asthma, with 25,000 individuals between the ages of 5 and 34 dying annually. More than 80% of deaths from asthma happen in low- and middle-income nations. Alarmingly, asthma prevalence is expected to surge by 100 million by 2025. This long-term illness severely reduces social skills, academic proficiency, and school performance [3]. The Worldwide Illness Burden report measures asthma's impact using Disability Adjusted Life Years, ranking it second alongside other chronic diseases like Alzheimer's and diabetes, and emphasizing the urgent need for effective management and prevention strategies [3].

Based on yearly hospitalizations of almost 500,000 people, asthma has a major worldwide impact, according to the American Academy of Allergy, Asthma, and Immunology. Of the reasons why children under the age of 15 are hospitalized, asthma ranks third. In India, children aged 5-14 comprise a quarter of the population, with bronchial asthma prevalence ranging from 4% to 20% among school-going children across various regions [2].

Although asthma cannot be totally cured, it can be effectively controlled. Maintaining normal activity levels and lung function, preventing chronic symptoms and repeated exacerbations, and offering the best pharmacological therapy with the fewest possible side effects are the key objectives of managing asthma. Additionally, by supporting social adaptations within the family, school, and community and promoting involvement in sports and leisure activities, good management helps kids lead normal, happy lives [4].

Beyond just physical symptoms, a child's quality of life (QoL) can be greatly impacted by having chronic asthma. Fear of exacerbations and feelings of being "different" can erode confidence and sense of control, leading to emotional struggles. Fatigue, moodiness, and withdrawal often correlate with increased school absences, compromising academic performance. The constant threat of asthma attacks and activity limitations can foster anxiety, affecting a child's ability to participate fully in life. This emotional toll can be just as debilitating as the physical symptoms, emphasizing the need for comprehensive support to address the whole child's well-being [5].

Through education, breathing exercises, and support, kids who suffer from asthma may gain a sense of control and master their disease, reducing panic during acute episodes. Accurate evaluation of symptoms and improvement of QoL help children cope with their condition and feel less different from peers. As school-age children develop cognitively, they can begin taking responsibility for asthma management with parental guidance, fostering increased control, improved disease management, and enhanced QoL. Transferring control to the child is a crucial developmental process, promoting autonomy and confidence in managing their asthma, and ultimately empowering them to take charge of their health [5]. The prevalence of asthma was considerably greater in people who had a pet at home, used smoke-producing fuel there, had family members who had previously smoked, and were older by birth. Of those with asthma today, 72.3% reported these factors [6]. Despite its widespread recognition in Western medical literature, the Asthma Education Program (AEP) remains not enough in India, where it is rarely discussed or implemented. This significant gap highlights the need for increased awareness and adoption of AEP in India to improve asthma management and patient outcomes [7].

Clinic visits, emergency room (ER) visits, and hospitalizations are all considerably decreased by appropriate asthma management. Programs that involve parents/guardians in addition to children are more effective at encouraging comprehensive asthma control than those that only target children [8]. Children's asthmatic QoL is directly correlated with asthma severity and control. Enhancing QoL is crucial for managing severe asthma effectively, promoting better overall well-being and symptom reduction [9]. QoL refers to an individual's or population's overall well-being, encompassing both positive and negative aspects of their physical, emotional, social, and environmental experiences at a particular point in time [10]. Fatigue, difficulty concentrating, and sleep difficulties can result from untreated asthma. It may also lead to missed work/school, financial strain, emergency hospital visits, and, in severe cases, death. Effective management is crucial to prevent these consequences [11]. Exercise plays a crucial role in overall health and, surprisingly, also helps manage airway inflammation, benefiting individuals with respiratory conditions like asthma [12].

The entire interdisciplinary healthcare team, comprising physicians, specialists, nurses, pharmacists, mental health experts, therapists, and ancillary personnel, must prioritize the concept of QoL. It is critical to understand that therapy targets the patient as an individual, not just pathology or lab findings. Therefore, therapeutic objectives should balance medical needs with QoL considerations, focusing on outcomes that enhance overall well-being [10].

The study aimed to assess the efficacy of a comprehensive interventional package that includes Buteyko breathing exercises and health education on QoL among asthmatic children. We hypothesized that children who receive education on asthma and breathing exercises will be better able to improve QoL.

## Materials and methods

This research project was a true experiment [13] in quantitative science. The study was conducted at the asthma clinic of a selected hospital, and the subjects were asthmatic children [14]. The study was performed over a period of 13 months, from February 11, 2023, to March 9, 2024. Ethical clearance and registration at CTRI (Reg. No. CTRI/2021/05/043488) were obtained.

The study included children between 10 and 17 years old and were able to understand and follow the comprehensive interventional package. The study excluded children who are not able to follow the intervention due to severe exacerbation of asthma and cognitive/developmentally disabled children.

Children with asthma who attended the asthma clinic satisfied the inclusion requirements and were randomized [15] into two groups using the coverslip method. They were included in the sample. A comprehensive package of interventions, comprising education on asthma [16] and Buteyko breathing exercises [17], was given to the experimental group. The control group did not reach out for intervention.

The study's sample size of 94 (47 in each group) was computed using the sample size equation for repeated measures analysis of variance (ANOVA).

N = (zα/2 + Zβ)^2 ^_x_SD^2 ^_x_2/d^2^

Here, zα/2 is the Z value at an α error, Zβ is the value at β error, SD is the average standard deviation of the character, calculated by SD1 + SD2/2, where SD1 and SD2 are the standard deviations of the character in each group, and d is the clinically relevant effect size.

**Figure 1:**
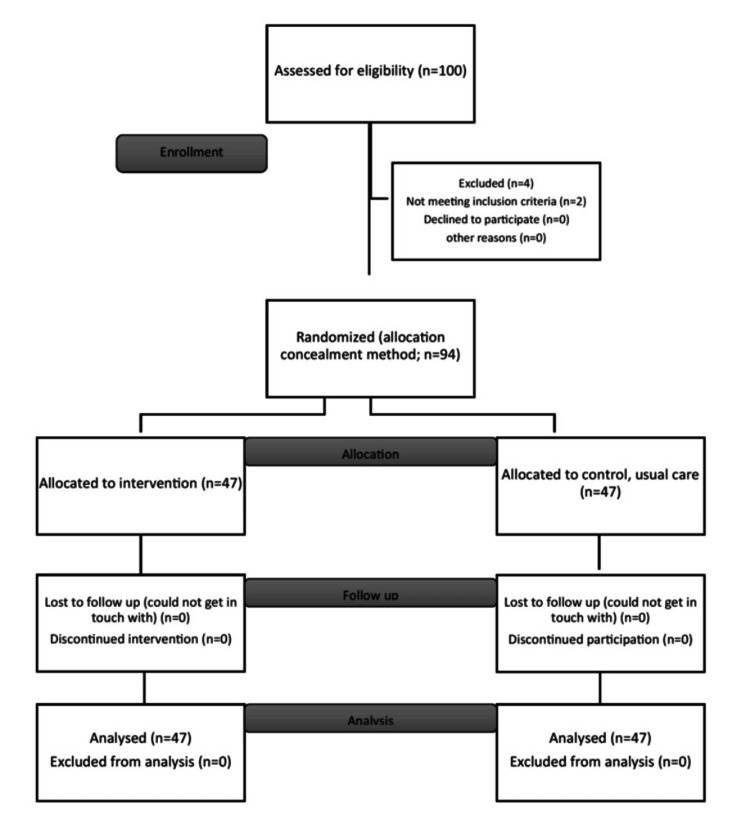
CONSORT flowchart CONSORT: Consolidated Standards of Reporting Trials

The average of two SD values (0.95 and 0.75) is 0.85. d is taken as 0.5, Zα = 1.96, Zβ = 0.84, and SD is 0.85 on average. To detect a difference of 0.5 asthma control as statistically significant between the two groups, the sample size required is 47. Therefore, 47 were in each group for the intervention part and in the control group. The total sample size is 94 (Figure 1).

Data from the sample were obtained for this study using three questionnaires: the Asthma Control Questionnaire (ACQ), the Mini Pediatric Asthma QoL Questionnaire (MiniPAQLQ), and an Asthma Knowledge Questionnaire (see the Appendix). The asthma knowledge questionnaire was divided into three sections: an assessment of respiratory outcomes such as oxygen saturation, breath holding time, respiratory rate, and PEFR was included in the third section, along with demographic data in the first section. Elizabeth F. Juniper created the standardized MiniPAQLQ. There were 13 questions total, broken down into three categories: emotional functions (four questions), symptoms (six questions), and activity constraints (three questions). It employs a 7-point Likert scale [18], where a score of 7 represents excellent health-related QoL and 1 represents poor health-related QoL for each question [19].There are six questions on the standard ACQ. Children use a 7-point rating system to answer each of the six questions about their experiences over the previous seven days (0 being completely uncontrollable to 6 being exceedingly regulated) [19].To track each subject's progress, the same tool was used four times in a span of four weeks.

Comprehensive interventional package

Health Education

For children with asthma, health education [20]is a methodically planned educational program. Definition, risk factors, causes, clinical symptoms, diagnosis, treatment, and prevention of bronchial asthma [21] were all covered in this lesson plan. Utilizing flash cards, the researcher instructed the experimental group's members.

The Buteyko Breathing Techniques [22] are a series of shallow breathing exercises that are useful in managing asthma symptoms. Konstantin Pavlovich Buteyko, a Ukrainian, is credited with creating the therapy's moniker. He developed its guiding principles in the 1950s. By teaching asthmatic patients how to hold their breath at functional residual capacity and stressing the value of mouth taping to raise arterial and alveolar carbon dioxide tension, this therapy tries to teach patients how to limit ventilation. It is suggested that the experimental group subjects practice three times a day for 15-20 minutes for a minimum of six weeks [23]. The Buteyko approach combines instruction and practice regarding the benefits of nasal breathing over oral breathing. The nose warms, filters, and humidifies inspired air in addition to producing nitric oxide, a powerful bronchodilator for asthma. Patients with Buteyko are advised to try to tap their mouth at night and to breathe through their noses during the day to promote nasal breathing. Beyond just breathing, the Buteyko approach suggests dietary adjustments, allergy avoidance, and stress management [24].Breathing exercises could be beneficial, especially for stress-induced asthma symptoms or asthma symptoms during a panic attack. They provide a method for calming down and concentrating on the breath. Breathing in and out while 10 steady breaths are counted [25].

Data collection process

Participants who met the inclusion criteria were randomly divided into two groups, each having 47 samples. Data collection took place between February 11, 2023, and March 09, 2024. Self-administered questionnaires for asthma knowledge, QoL, and symptom control [26] were made available by the investigator to gather data. After completing a questionnaire, the child's respiratory outcomes were evaluated and recorded, including oxygen saturation, peak expiratory flow rate (PEFR), respiratory rate, and breath-holding time. The investigator then educated the patient about asthma and showed the experimental group a basic breathing exercise technique called the Buteyko breathing exercise. The child was then asked to repeat the demonstration and perform it three times a day from home. Using the same instruments for tracking their development, data were gathered from the experimental group by the first, fourth, eighth, and twelve weeks.

## Results

Table 1 demonstrates that the majority of individuals in the control and experimental groups are between the ages of 10 and 13 years. The experimental group consisted of 26 (55%) female and 21 (45%)male asthmatic children, while the control group had 23 (49%) female and 24 (51%) male asthmatic children. Compared to 32 (68%)of the control group, 38 (83%) of the experimental group's asthmatic children were enrolled in upper primary school. While 21 (45%) of the participants in the experimental group were from rural areas, 20 (43%) participants in the control group were from rural areas. The child populations in both the experimental and control groups were predominantly Muslim. Participants with a family history of asthma made up 10 (21%) of the experimental group and 15 (32%) of the control group. In both groups, most patients had asthma for six to ten years. In the experimental group, 47 (100%) of participants were using asthma medications for symptom control, and 45 (96%) participants were in the control group.

**Table 1:** Frequency distribution of background between the experimental and control groups UP: upper primary; HS: high school; HSS: higher secondary school

Demographic characteristics		Experimental group (N = 47)		Control group (N = 47)		Total (N = 97)	
		Frequency (N)	Percentage (%)	Frequency (N)	Percentage (%)	Frequency (N)	Percentage (%)
Age in years	10-13	40	85	35	74	97	100
	14-17	7	15	12	26		
Sex	Male	21	45	24	51	97	100
	Female	26	55	23	49		
Education	UP	38	83	32	68	97	100
	HS	6	13	13	28		
	HSS	3	6	2	4		
Residential area	Urban	26	55	27	57	97	100
	Rural	21	45	20	43		
Religion	Hindu	11	24	15	32	97	100
	Muslim	33	70	27	57		
	Christian	3	6	5	11		
Family history of asthma	Yes	10	21	15	32	97	100
	No	37	79	32	68		
Time since diagnosis (in years)	<1	1	2	2	4	97	100
	1-5	8	17	12	26		
	6-10	35	75	32	68		
	11-15	3	6	1	2		
Usage of asthma medicine	No	0	0.00	2	4	97	100
	Yes	47	100	45	96		

The mean knowledge levels of the experimental and control groups at various time points differ significantly, as demonstrated in Table 2. When comparing the effectiveness of the control group and the experimental group, the experimental group's mean knowledge level increased from the first to the fourth visit (mean value of the first visit: 10.15, mean value of the fourth visit: 20.51), and there was a significant group difference (F value = 160) between the two groups. The significant difference between the control and experimental groups at various time points was ascertained using an ANOVA repeated measures test. The comparison reveals that at certain points in time, both groups changed significantly (p < 0.001). Therefore, raising knowledge levels was a successful outcome of the whole interventional package.

**Table 2:** Analysis of effectiveness of comprehensive interventional package on level of knowledge

Groups	Knowledge level, mean (SD)					
	Pretest	Posttest-1	Posttest-2	Posttest-3	F-Value	p value
Control group, n = 47	11.38 (3.97)	12.72 (3.993)	13.11 (3.84)	14.15 (4.42)	160.293	<0.001
Experimental group, n = 47	10.15 (4.72)	23.23 (4.16)	26.36 (4.73)	26.87 (5.07)		
Total	10.77 (4.38)	17.98 (6.66)	19.73 (7.92)	20.51 (7.96)		

Table 3 shows that the experimental group's mean QoL increased from 3.13 to 5, suggesting that they had a higher QoL related to their health than the control group. With a p value of less than 0.001, this improvement was statistically significant and demonstrated the effectiveness of the interventional package in raising the QoL for children with asthma.

**Table 3:** Effectiveness of comprehensive interventional package on quality of life. Likert scale with maximum possible score for each item is 7 (good health-related quality of life) and the minimum score is 1 (poor health-related quality of life) SD: standard deviation

Groups	Pediatric asthma quality of life, mean (SD)					
	Pretest	Posttest-1	Posttest-2	Posttest-3	F value	p value
Control group, n = 47	3.24 (0.39)	3.29 (0.399)	3.38 (0.37)	3.40 (0.398)	490.92	<0.001
Experimental group, n = 47	3.13 (0.32)	4.05 (0.37)	4.75 (0.28)	5 (0.32)		
Total	3.18 (0.36)	3.67 (0.54)	4.07 (0.76)	4.19 (0.87)		

Table 4 demonstrates variation between the groups due to the extensive asthma management intervention package (F value = 370.4). The experimental group showed considerably improved control (p value of 0.001) in terms of asthma management compared to the control group. This suggests that better asthma management was achieved by health education and breathing exercises.

**Table 4:** Effectiveness of interventional package on asthma control. Children recall their experiences during the previous seven days and respond to each of the six questions using a 7-point scale (0 = totally controlled to 6 = extremely controlled) SD: standard deviation; ACQ: asthma control questionnaire

Groups	Asthma control, mean (SD)					
	Pretest	Posttest-1	Posttest-2	Posttest-3	F value	p value
Control group, n = 47	3.59 (0.499)	3.52 (0.37)	3.35 (0.39)	3.39 (0.396)	370.4	<0.001
Experimental group, n = 47	3.71 (0.48)	2.92 (0.54)	1.79 (0.36)	1.2 (0.43)		
Total	3.65 (0.49)	3.22 (0.55)	2.57 (0.87)	2.29 (1.18)		

## Discussion

In this study, most participants belonged to the age category of 10-13 years. There were 26 (55%) female and 21 (45%) male asthmatic youngsters in the experimental group. There were 23 (49%) female and 24 (51%) male children with asthma in the control group. Compared to 32 (68%) in the control group, 38 (83%) of the asthmatic children in the experimental group were enrolled in upper primary school. The youngsters in both the experimental and control groups were predominantly urban Muslim individuals. While 15 (32%) of individuals in the control group had a family history of asthma, 10 (21%) of those in the experimental group did. The majority of individuals in the control and experimental groups suffered from asthma for six to ten years. Asthma medicine was taken by 47 (100%) individuals in the experimental group and 45 (96%) individuals in the control group.

Analysis of the comprehensive interventional package's effectiveness

At a p value of less than 0.001, the comprehensive interventional package's impact on asthmatic children's knowledge level was significant. This suggests that the control and experimental groups had significantly different knowledge levels. An asthmatic child's understanding of their condition is essential for fostering a positive attitude toward asthma therapy, recognizing symptoms, and responding correctly for efficient disease management, asthma control, and a high QoL [27], research contrasting the effects of an intensive AEP (group B) with a regular AEP (group A). A prospective, randomized, single-blind trial was conducted in a Hong Kong public hospital's pediatric department. Children with acute asthma attacks, aged 2-15 years, who were admitted to the pediatric department, were recruited. The primary end outcomes are the frequency of ER visits and hospital admissions for asthma throughout the three-month follow-up period. Group B had a statistically significant decrease in emergency department visits and hospitalizations. Group B also saw a notable improvement in drug compliance [28].

In contrast to the group under control, the experimental group's QoL significantly improved. With a p value of less than 0.001, the extensive interventional package, including breathing exercises and health education, was significant. Thus, this intervention would enhance the QoL associated with excellent health for children with asthma. Even if there is a safe and effective treatment for bronchial asthma, children's QoL in relation to their health is significantly impacted. A research project assessed the QoL of children with bronchial asthma to ascertain the effects of various clinical and sociodemographic factors on this QoL. A tertiary care teaching hospital's pediatric chest clinic conducted a study on children aged 7-17 years old using the Standardized Pediatric Asthma QoL Questionnaire, PAQLQ(S). Children's PAQLQ(S) scores showed greater impairment if symptoms started before the age of a year if they experienced frequent exacerbations, poor treatment compliance, poorly controlled symptoms, or if they had a history of missing school. The emotional function mean PAQLQ(S) score was the lowest among newly diagnosed and follow-up cases. There was a statistically significant difference between the PAQLQ(S) scores of asthma cases that were controlled, partially controlled, and uncontrolled [29].

This study shows that controlling asthma was successfully achieved with the whole interventional package. The results of the repeated measures ANOVA indicate a statistically significant difference between the asthma control scores at different time periods, with a p value of less than 0.001. When the two groups' asthma control was compared, the experimental group's asthma control was superior to that of the control group. A study was conducted to find out how well the Buteyko Method works to improve school-age children's QoL and asthma management. The analysis included 14 individuals, aged 7-11, who had been diagnosed with bronchial asthma. They were split evenly into two groups: the experimental group was required to attend lectures and demonstrations of the Buteyko Method, while the control group did not receive any treatment. For three weeks in a row, the experimental group was visited to assess compliance and progress. Additionally, before the start of the intervention phase and once a week throughout the follow-up appointments, each group was requested to complete the Pediatric Asthma QoL Questionnaire and the ACQ pre- and postintervention questionnaires. The experimental group demonstrated a notable distinction following the management of the Buteyko Method (p = 0.002), but no significant difference was seen (p = 0.177) between the asthma control pretest and posttest mean scores of the control group. Conversely, the control group's QoL pre- and posttest mean ratings did not significantly differ in any week during the one-month follow-up period (p = 0.736, 0.604, and 0.689). Conversely, the experimental group demonstrated no significant difference on the second visit (p = 0.111) but a significant difference on the third (p = 0.035) and fourth (p = 0.002) visits. Using the Buteyko Method in conjunction with traditional asthma care during a three-to-four-week timeframe helps enhance asthma control and school-age children's QoL [30].

The interventional package improved QoL, asthma knowledge, and symptom management among asthmatic children, leading to better overall health outcomes. This study findings helps for prevention and promotive strategies to be adopted to manage the asthmatic children.

The study's scope was limited to children aged 10-17 years, excluding parental involvement.

## Conclusions

The comprehensive interventional package, which includes health education about asthma and Buteyko Breathing Exercises, was effective in improving knowledge and asthma control, as well as improving their QoL. Along with routine medical treatment, health education and breathing exercises can help children better control their asthma and enhance their QoL.

## Appendices

**Table 5:** Asthma knowledge questionnaire

Sl. no.	Questions	Answers		
Section A: Etiology				
1	Asthma symptoms can be caused by			
a	Allergy	Yes	No	Can’t say
b	Air pollution or any other type of irritant (dust, fumes, etc.)	Yes	No	Can’t say
c	Living with a person who has a common cold	Yes	No	Can’t say
d	Common cold	Yes	No	Can’t say
e	Exercise	Yes	No	Can’t say
f	Certain foods	Yes	No	Can’t say
g	Certain drugs	Yes	No	Can’t say
h	Without obvious reason	Yes	No	Can’t say
2	I can pass my asthma to a person I am living with	Yes	No	Can’t say
3	Asthma is more likely if a blood relative in the family or in a previous generation already has asthma	Yes	No	Can’t say
Section B: Pathophysiology				
4	In asthma, the breathing tubes in the lungs become narrow due to swelling of their walls	Yes	No	Can’t say
5	In asthma, the breathing tubes also become narrow due to the tightening of muscles around them	Yes	No	Can’t say
6	In asthma, the breathing tubes also become narrow due to mucous (sputum) collection	Yes	No	Can’t say
Section C: Symptoms and assessment of severity				
7	Symptoms of asthma are breathing difficulty with wheezing or whistling sound	Yes	No	Can’t say
8	Asthma symptoms vary in severity from time to time, being less sometimes and more at other times	Yes	No	Can’t say
9	Asthma symptoms are more likely to occur at night or early morning	Yes	No	Can’t say
10	I can judge how severe my asthma is	Yes	No	Can’t say
11	Severity of asthma can be measured by a test of blowing out air into a machine	Yes	No	Can’t say
12	Severity of asthma can also be measured at home using a simple device called a peak flow meter	Yes	No	Can’t say
13	Asthma attacks can be dangerous	Yes	No	Can’t say
14	I can interpret the symptoms of asthma that worsen my condition	Yes	No	Can’t say
Section D: Medication				
15	Asthma medicines can be given as tablets/syrups/inhalers	Yes	No	Can’t say
16	The best way to take asthma medicine is by inhalation	Yes	No	Can’t say
17	Asthma medicines are usually of two types: one to give immediate relief from symptoms (relievers) and the other to prevent symptoms (preventers)	Yes	No	Can’t say
18	I know which is the drug for regular use and which is to be used if breathlessness occurs	Yes	No	Can’t say
19	The most effective drugs for controlling asthma are called steroids	Yes	No	Can’t say
20	Steroids can be harmful, but given by inhalers, they are usually free from significant harmful effects	Yes	No	Can’t say
21	Medicine for asthma has to be taken until my symptoms persist and then can be stopped	Yes	No	Can’t say
22	Medicine for asthma has to be taken even after symptoms are no longer there until my doctor advises me to	Yes	No	Can’t say
Section E: Preventive				
23	If I can identify and avoid factors that increase my symptoms, my asthma will be controlled better	Yes	No	Can’t say
24	I can prevent or reduce asthma symptoms by taking the reliever inhalers just before exposure to anything that triggers the symptoms	Yes	No	Can’t say
25	I can prevent asthma symptoms if I take my medication regularly	Yes	No	Can’t say
26	If the asthma symptoms are getting worse, I know how to change my medication	Yes	No	Can’t say
Section F: Natural history				
27	Asthma cannot be cured	Yes	No	Can’t say
28	Although asthma cannot be cured, it can usually be controlled fairly well	Yes	No	Can’t say

**Table 6:** Mini pediatric asthma quality of life questionnaire

Sl. no.	Questions	Responses			
		First visit score	Second visit score	Third visit score	Fourth visit score
1	How much did COUGHING bother you in the past seven days? (BLUE CARD)	-	-	-	-
2	How much did WHEEZING (NOISY BREATHING) bother you during the past seven days? (BLUE CARD)	-	-	-	-
3	How much did TIGHTNESS IN YOUR CHEST bother you during the past days? (BLUE CARD)	-	-	-	-
4	How often did you feel DIFFICULTY IN BREATHING during the past seven days? (GREEN CARD)	-	-	-	-
5	How often did your asthma make you feel TIRED during the past seven days? (GREEN CARD)	-	-	-	-
6	How often did you have trouble SLEEPING AT NIGHT because of your asthma during the past seven days? (GREEN CARD)	-	-	-	-
7	How often did your asthma make you feel DISAPPOINTED during the seven days? (GREEN CARD)	-	-	-	-
8	How often did you feel VERY AFRAID OR WORRIED because of your asthma during the past seven days? (GREEN CARD)	-	-	-	-
9	How often did your asthma make you feel SHORT-TEMPERED during the past seven days?	-	-	-	-
10	How often did you feel DIFFERENT OR LEFT OUT because of your asthma during the past seven days? (GREEN CARD)	-	-	-	-
11	How much have you been bothered by your asthma in PHYSICAL ACTIVITIES (such as running. swimming. sports, walking uphill upstairs, and bicycling) during the past seven days? (BLUE CARD)	-	-	-	-
12	How much have you been bothered by your asthma in BEING WITH ANIMALS (such as playing with pets and looking after animals) during the past seven days? (BLUE CARD)	-	-	-	-
13	How much have you been bothered by your asthma in ACTIVITIES WITH FRIENDS AND FAMILY (such as playing at recess and doing things with your friends and family) during the past seven days? (BLUE CARD)	-	-	-	-

**Table 7:** Asthma control questionnaire

Sl. No.	Questions	Responses
1	On average, during the past seven days, how often were you woken by your asthma during the night?	0: Never; 1: Hardly ever; 2: A few times; 3: Several times; 4: Many times; 5: A great many times; 6: Unable to sleep because of asthma
2	On average, during the past seven days. How bad were your asthma symptoms when you woke up in the morning?	0: No symptoms; 1: Very mild symptoms; 2: Mild symptoms; 3: Moderate symptoms; 4: Somewhat severe symptoms; 5: Severe symptoms; 6: Very severe symptoms
3	In general, during the past seven days, how limited were you in your activities because of your asthma?	0: Not limited at all; 1: Very slightly limited; 2: Slightly limited; 3: Moderately limited; 4: Extremely limited; 5: Very limited; 6: Totally limited
4	In general, during the past seven days, how much shortness of breath did you experience because of your asthma?	0: None; 1: A very little; 2: A little; 3: A moderate amount; 4: Quite a lot; 5: A great deal; 6: A very great deal
5	In general, during the past seven days, how much time did you wheeze?	0: Never; 1: Hardly any of the time; 2: A little of the time; 3: A moderate amount of the time; 4: A lot of the time; 5: Most of the time; 6: All the time
6	On average, during the past seven days, how many puffs/inhalations of short-acting bronchodilators (e.g., Asthalin/Ventolin) have you used each day? (if you are not sure how to answer this question, please ask for help)	0: None; 1: 1-2 puffs/inhalations most days; 2: 3-4 puffs/inhalations most days; 3: 5-8 puffs/inhalations most days; 4: 9-12 puffs/inhalations most days; 5: 13-16 puffs/inhalations most days; 6: More than 16 puffs/inhalations most days
